# Draft Genome Sequences of 13 Vibrio cholerae Strains from the Rio Grande Delta

**DOI:** 10.1128/MRA.00308-21

**Published:** 2021-06-03

**Authors:** Jeffrey W. Turner, Jorge Duran-Gonzalez, David A. Laughlin, Daniel Unterweger, David Silva, Boris Ermolinsky, Stefan Pukatzki, Daniele Provenzano

**Affiliations:** a Department of Life Sciences, Texas A&M Corpus Christi, Corpus Christi, Texas, USA; b Department of Biology, University of Texas Rio Grande Valley, Brownsville, Texas, USA; c Max Planck Institute for Evolutionary Biology, Plön, Germany; d Institute for Experimental Medicine, Kiel University, Kiel, Germany; e Department of Biology, The City College of New York, New York, New York, USA; University of Southern California

## Abstract

Vibrio cholerae is the etiologic agent of cholera, an acute and often fatal diarrheal disease that affects millions globally. We report the draft genome sequences of 13 non-O1/O139 V. cholerae strains isolated from the Rio Grande Delta in Texas. These genomes will aid future analyses of environmental serovars.

## ANNOUNCEMENT

Vibrio cholerae is a Gram-negative curved bacterium that thrives in tropical and temperate aquatic ecosystems (1). The species was first identified as the cause of cholera by the Italian physician Filippo Pacini in 1854; the German physician and bacteriologist Robert Koch independently confirmed this discovery in 1883 (2). Toxigenic O1 serovar strains are responsible for pandemic disease outbreaks (3). In 1993, a subgroup of O1 strains converted to the O139 serogroup and caused local outbreaks but did not become pandemic (4). Additional serovars are commonly isolated from cholera patients (5), and a global increase in non-O1/O139 infections has been linked to climate change (6).

V. cholerae strains were isolated from plankton samples obtained from the following two sampling sites on the Rio Grande Delta along the Mexico-United States border where the cities of Matamoros and Brownsville form a transborder agglomeration: sites 21 (25°53′56.54″N, 97°29′52.09″W) and 42 (25°57′17.58″N, 97°08′44.42″W). Isolation was achieved by culture on thiosulfate-citrate-bile salts-sucrose (TCBS) agar plates (Becton, Dickinson, Franklin Lakes, NJ) incubated overnight at 30°C as described earlier (7). Genomic DNA was isolated from sucrose-fermenting CFUs by sodium dodecyl sulfate (SDS) solubilization and phenol-chloroform extraction. Amplification of the 16S-23S rRNA intergenic spacer region using the prVC-F and prVCM-R primers (8) was used for typing. Serogrouping was performed by the National Institute of Infectious Diseases in Tokyo, Japan, as described elsewhere (9). Sequencing libraries (100-bp paired-end format) were prepared using the TruSeq DNA library prep kit (Illumina, San Diego, CA, USA). An Agilent 2100 Bioanalyzer (Santa Clara, CA, USA) was used to determine the library size and concentration. Sequencing was completed by Ambry Genetics Corporation (Aliso Viejo, CA, USA) using an Illumina HiSeq 2000 device. The raw sequence reads were inspected for quality using FastQC version 0.11.5 (10) to inform the genome assembly settings. The draft genome sequences were assembled *de novo* using Edena version 3.131028 (11) with default settings, with the following two exceptions: the 3′ ends were truncated to remove the low-quality bases (option -t 10), and the minimum contig size was set at 500 bp (option -c 500). The Edena assembler features exact read matching and spurious read removal that obviates read preprocessing when working with HiSeq 2000 data. Annotation was completed using the Prokaryotic Genome Annotation Pipeline (PGAP) version 3.2 (12). Table 1 provides the accession numbers and general metrics of each assembly as well as the serogroup of each isolate. The draft genome sequences for DL4211 and DL4215 were described previously (13); however, those genomes were replaced in DDBJ/ENA/GenBank with the higher-quality assemblies described here.

**TABLE 1:** Accession numbers, genome assembly metrics, and serogroups of the 13 V. cholerae isolates from the Rio Grande Delta

Strain	GenBank accession no.	SRA accession no.	No. of reads	Coverage (×)^*a*^	No. of contigs	*N*_50_ (bp)	GC content (%)	Size (bp)	Serogroup^*b*^
DL2111	MSSN00000000	SRR14319855	12,939,391	113.8	88	189,346	47.4	4,119,702	Rough
DL2112	MSSO00000000	SRR14319854	6,980,134	64.0	107	123,726	47.4	4,118,072	Rough
DL2113	MSSP00000000	SRR14319850	5,802,868	52.9	118	79,342	47.4	4,114,718	Rough
DL2114	MSSQ00000000	SRR14319849	7,733,503	69.8	85	231,987	47.4	4,113,495	O74
DL2115	MSSR00000000	SRR14319848	9,312,550	82.5	97	164,794	47.4	4,119,886	Rough
DL2116	MSSS00000000	SRR14319847	11,999,442	107.3	99	150,335	47.4	4,120,548	Rough
DL2117	MSST00000000	SRR14319846	9,438,288	83.8	92	160,631	47.4	4,122,195	Rough
DL4211	MSSU00000000	SRR14319845	51,281,125	441.3	113	200,733	47.5	4,043,910	O123
DL4212	MSTE00000000	SRR14319844	29,518,632	255.2	71	195,524	47.4	4,115,192	O4
DL4213	MSTX00000000	SRR14319843	9,241,571	82.5	78	269,860	47.6	4,031,163	O109
DL4214	MSTF00000000	SRR14319853	4,774,116	42.3	95	137,521	47.4	4,100,910	O4
DL4215	MSTG00000000	SRR14319852	46,233,778	412.7	67	205,170	47.5	4,005,821	O113
DL4216	MSTH00000000	SRR14319851	11,046,830	96.3	83	186,400	47.5	4,110,306	O26

a Coverage refers to the minimum required contig coverage set automatically by the assembler.

b The rough designation describes isolates devoid of O antigen (9).

### Data availability.

This whole-genome shotgun project has been deposited at DDBJ/ENA/GenBank under the accession numbers listed in Table 1. The 13 genome assemblies were organized under BioProject accession number PRJNA359496.
